# Pharmacokinetics of gentamicin and vancomycin during continuous venovenous hemofiltration in critically ill septic patients with acute kidney injury

**DOI:** 10.1186/cc12374

**Published:** 2013-03-19

**Authors:** N Petejova, J Duricova, A Martinek, J Zahalkova

**Affiliations:** 1 University Hospital, Ostrava, Czech Republic; 2 Hospital, Sternberk, Czech Republic

## Introduction

Acute kidney injury (AKI) is a common complication of critical illness and sepsis [1]. Dosing of antibacterial agents in septic patients is complicated by altered pharmacokinetics due to both acute renal failure and critical illness [2]. Current dosing regimens for administration of gentamicin and vancomycin to septic patients with AKI on continuous venovenous hemofiltration (CVVH) at a filtration rate of 45 ml/kg/hour are missing.

## Methods

Seventeen septic patients with AKI treated with vancomycin and seven patients with gentamicin on CVVH were included. In the vancomycin group, patients received the first dose of 1.0 g intravenously followed by 1.0 g/12 hours if not adjusted. In the gentamicin group, patients received a loading dose of 240 mg followed by a maintenance dose every 24 hours. The vancomycin maintenance dose was optimized to achieve AUC_0-24_/MIC ≥400 (Cmin >10 mg/l), gentamicin target was Cmax/MIC of 8 to 10. Maintenance doses were adjusted according to drug level simulation using a pharmacokinetic programme.

## Results

The median vancomycin total clearance (Cltot) was 0.89 and 0.55 ml/minute/kg on the first and second day of the study. CRRT clearance accounted for about 50 to 60% of vancomycin Cltot found in a population with normal renal function (0.97 ml/minute/kg). Vancomycin serum concentrations after the first dose were below the required target of 10 mg/l as early as 6 hours in 10 patients. AUC_0-24_/MIC ≥400 ratio was achieved in 67% of patients on the first day. The median gentamicin Cltot was 0.68 and 0.793 ml/minute/kg on the first and second day of the study. CRRT clearance accounted for about 50% of gentamicin Cltot found in a population without renal impairment (0.73 ml/minute/kg). The target Cmax/MIC ratio was achieved in 78% of patients after the first dose.

## Conclusion

CVVH at a filtration rate of 45 ml/kg/hour leads to high removal of both antibiotics. Due to rapid change in patient's clinical status it was impossible to predict a fixed dosage regimen. We recommend administration of unreduced loading dose and: blood sampling as early as 6 hours after first vancomycin dose; blood sampling 30 to 60 minutes after gentamicin administration and before the next dose; and the maintenance dose should be based on drug-level monitoring.
